# Obstructive jaundice in a patient with polycystic liver disease complicated with polycystic kidney and polycystic lung: A case report

**DOI:** 10.1097/MD.0000000000019511

**Published:** 2020-04-03

**Authors:** Liling Zhang, Linwang Gan, Qi Liu, Ying Li, Jiaru Lin, Santao Ou

**Affiliations:** Department of nephropathy, The First Affiliated Hospital of Southwest Medical University. Luzhou, Sichuan, China.

**Keywords:** obstructive jaundice, polycystic kidney disease, polycystic liver disease, polycystic lung

## Abstract

**Rationale:**

Polycystic liver disease (PLD) is an autosomal-dominant disorder that is commonly associated with autosomal-dominant polycystic kidney disease (PKD) but rarely complicated with polycystic lung. Here, we report the first case of severe obstructive jaundice caused by multiple liver cysts in a patient with PLD complicated by PKD and polycystic lung.

**Patient concerns:**

A 72-year-old man with a history of PLD complicated with polycystic kidney presented with progressive jaundice, hematuria, poor appetite, nausea, and weight loss since 3 months.

**Diagnosis:**

PLD complicated with PKD and polycystic lung was identified using computed tomography, and obstructive jaundice was identified using magnetic resonance imaging and magnetic resonance cholangiopancreatography.

**Interventions:**

The patient could not undergo surgery, and was therefore treated with combined bilirubin adsorption and continuous veno-venous hemofiltration.

**Outcomes:**

The patient's symptoms and laboratory findings improved after bilirubin adsorption and continuous veno-venous hemofiltration. Unfortunately, the patient was unable to continue the treatment due to financial reasons, and died of shock most likely due to cyst rupture.

**Lessons:**

Imaging examination of the lungs is necessary for patients with PLD. Although infrequent, jaundice can occur in these patients and cause severe hyperbilirubinemia. When surgery is contraindicated, blood purification may serve as an alternative treatment for patients with PLD-related obstructive jaundice.

## 1 Introduction

Polycystic liver disease (PLD) is a rare genetic disease caused by mutations in the *PKD1*, *PKD2*, or *PRKCSH* gene.^[1]^ The characteristic manifestation of PLD is liver cysts that originate from bile-duct epithelial cells owing to PLD-related gene mutations. As the liver cysts grow, the hepatic volume increases at a rate of 0.9% to 1.6% per year; in some patients, the total hepatic volume can exceed 10 L.^[2]^ The symptoms of PLD are non-specific and are mainly attributable the increase in hepatic volume; they include indigestion, loss of appetite, nausea, vomiting, and right upper abdominal pain. Obstructive jaundice is a rare manifestation of PLD, and is usually a consequence of bile-duct compression by external cysts, especially, in patients with numerous hepatic cysts.^[3,4]^

PLD can not only occur alone as an autosomal-dominant genetic anomaly, but it can also be accompanied by autosomal-dominant or autosomal-recessive polycystic kidney disease (PKD).^[5,6]^ However, the combination of PLD with polycystic lung is rare. In this report, we present an extremely rare case of obstructive jaundice in a patient with PLD complicated by PKD as well as polycystic lung.

## 2 Case report

A 72-year-old man was admitted to our hospital due to the rupture of some renal cysts. He had first been admitted to our hospital 3 years ago because of hematuria associated with PLD complicated with PKD and secondary renal dysfunction (estimated glomerular filtration rate: 40 mL/minute/1.73 m^2^). At that time, hemostasis was successfully achieved, and the hematuria disappeared. The patient was discharged and advised to take detoxification drugs, including Niaoduqing particles and activated charcoal tablets. He was followed up at our hospital for 1 year, during which time his renal function was stable; however, he stopped attending the follow-up exams after 1 year. At 3 months before the present hospitalization, he developed hematuria again, followed by progressive jaundice, loss of appetite, nausea, and weight loss. He had a family history of PKD; his 2 sons have PLD complicated with PKD.

A physical examination during the current hospitalization revealed severe and extensive yellowing of the skin and sclera, and a large, non-tender abdominal mass, with no signs of chronic liver disease, such as ascites and splenomegaly. The results of liver-function tests were indicative of obstructive jaundice: total bilirubin, 12.29 mg/dL (normal range, <1 mg/dL); direct bilirubin, 9.22 mg/dL (normal range, <0.4 mg/dL); alkaline phosphatase, 198.4 U/L (normal range, 45–125 U/L), γ-glutamyl transferase, 180.2 U/L (normal range, 10–60 U/L), aspartate transaminase, 45.2 U/L (normal range, 15–40 U/L), and serum creatinine, 7.69 mg/dL (normal range, 0.67–1.18 mg/dL). Additionally, serological tests for hepatitis were negative.

**Figure 1:**
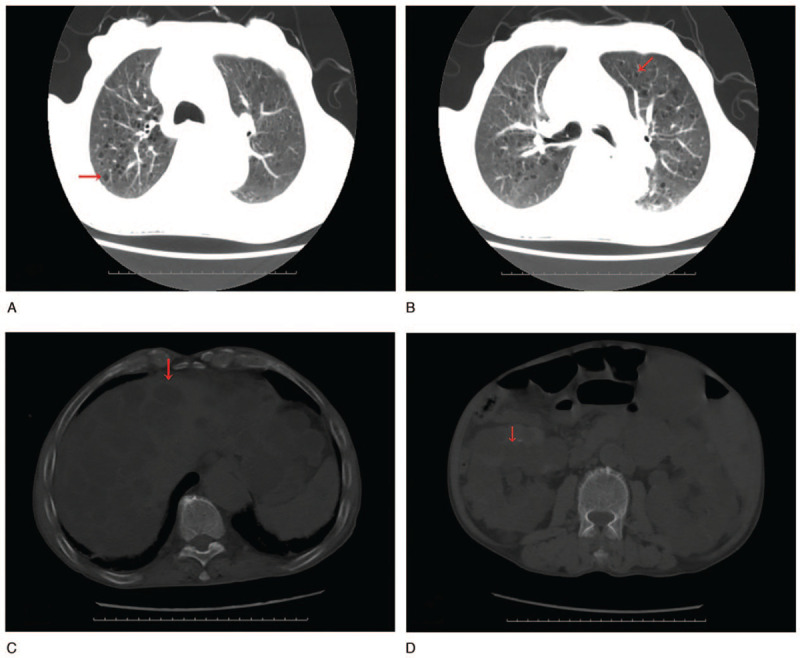
Computed tomography scans show multiple cysts (arrows) in the lungs, liver, and kidneys, with a very small proportion of normal residual parenchyma.

**Figure 2:**
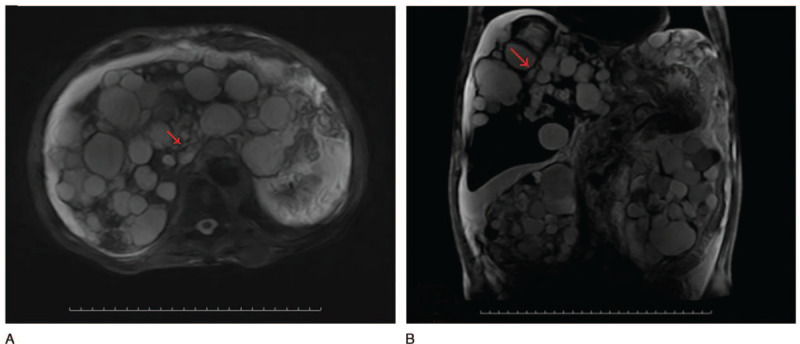
Magnetic resonance cholangiopancreatography revealed mild intrahepatic bile-duct expansion (arrows) and multiple hepatic and renal cysts.

Computed tomography (CT) demonstrated numerous cysts in the liver and kidneys with a very small proportion of normal residual parenchyma, and also revealed numerous cysts in the lungs (Fig. 1A–D). The findings of magnetic resonance imaging (MRI) and magnetic resonance cholangiopancreatography (MRCP) were indicative of obstructive jaundice resulting from the repression of numerous liver cysts, especially, 2 large cysts in the liver (Fig. 2A-B).

Because of the diffuse distribution of cysts in the liver and the severe renal dysfunction, the patient could not undergo surgical treatments such as percutaneous drainage of hilar cysts, open or laparoscopic cyst fenestration, combined hepatic resection and fenestration, and liver transplantation. Furthermore, medical treatment failed to improve the patient's jaundice. As the excessive bilirubin could have led to life-threatening bilirubin encephalopathy, we decided to perform dialysis to remove bilirubin from the circulation and relieve the symptoms. Bilirubin adsorption and continuous veno-venous hemofiltration (CVVH) were selected to ameliorate the hyperbilirubinemia and uremia. Surprisingly, the serum bilirubin level dropped from 12.29 mg/dL to 5.01 mg/dL and the levels of urea and creatinine decreased after a single session of combined CVVH-bilirubin adsorption treatment. However, the patient and his family chose hemoperfusion and hemofiltration for further treatment, as this combination was less expensive, though also less effective, than combined CVVH-bilirubin adsorption treatment. The patient has provided informed consent for the publication of this case report.

Unfortunately, 4 days after being discharged, the patient developed sudden shock and unconsciousness, which led to his demise. The sudden deterioration of the patient's condition may be attributable to spontaneous cyst rupture and bleeding.

## 3 Discussion

We have reported an extremely rare case of PLD complicated with PKD, polycystic lung, and obstructive jaundice in a 72-year-old man. The results of liver-function tests in our patient indicated obstructive jaundice: total bilirubin, 12.29 mg/dL and direct bilirubin, 9.22 mg/dL. MRI also suggested dilatation of the bile ducts adjacent to 2 large cysts, with no stones, sludge, or other lesions. Obstructive jaundice in a patient with liver cysts is not necessarily due to extrinsic compression of the bile ducts due to the cysts, but may be secondary to congenital cystic malformation of the ducts.^[7]^ Khoonsari et al^[8]^ and Dumonta et al^[9]^ each reported a case of obstructive jaundice secondary to compression of liver cysts. In both cases, exploratory laparotomy and surgical decompression were performed. Unfortunately, our patient could not undergo surgery due to multiple liver cysts of varying sizes and severe renal failure. Although initial treatment with bilirubin adsorption-CVVH dramatically reduced the serum bilirubin level, the patient was unable to continue this treatment due to financial reasons. He eventually succumbed to his disease, and the final cause of death was most likely shock due to cyst rupture and hemorrhage.

PLD is a rare genetic disease characterized by the development of multiple hepatic cysts owing to the abnormal differentiation of the biliary ducts.^[10]^ PLD is classified into 3 types: autosomal dominant PLD, PLD combined autosomal-dominant PKD, and PLD combined with autosomal-recessive PKD. Polycystic lung may rarely coexist with PLD or autosomal-dominant PKD. Zhang et al have reported a case of PKD complicated with PLD and polycystic lung that presented with cough and hemoptysis.^[11]^ However, their patient did not develop obstructive jaundice and did not require hemodialysis. To our knowledge, we have reported the first case of PLD coexisting with PKD and polycystic lung that was complicated by obstructive jaundice and treated with bilirubin adsorption and CVVH.

PLD is more frequent in females than in males. In female patients with PLD, the hepatic cysts can markedly increase in size and number during childbearing age. Studies have shown that PLD is clinically silent in the early stage; however, the symptoms of persistent abdominal pain, abdominal distension, gastroesophageal reflux, back pain, and hepatic vein and portal vein compression appear in later stages, and severe complications such as cyst hemorrhage, rupture, and infection occur in nearly 50% of patients by the final stages.^[12]^ A fatal case of hepatic cyst rupture in a patient with PLD complicated with autosomal-dominant PKD has been reported in the literature.^[13]^ In our patient, the cause of death was most likely diffuse cyst hemorrhage in the liver or kidney.

Complications of advanced liver disease and liver failure rarely appear in patients with PLD, and the liver function is well preserved despite the development of portal hypertension and hepatosplenomegaly. Furthermore, the incidence of jaundice is quite low among PLD patients, unless concomitant choledocholithiasis or cholangiocarcinoma cause obstructive jaundice.^[11]^ In our patient, however, MRI and MRCP showed severe obstructive jaundice related to PLD.

The treatment strategy for the complication of polycystic kidney and polycystic lung is aimed at reducing the cysts or organ volume, relieving the symptoms, and improving the quality of life. To treat PKD, cell proliferation and cyst growth are inhibited using drugs and recently, using gene therapy.^[14]^ Laparoscopic surgical treatment is mainly used to decompress the cysts to reduce the development of renal failure.^[15]^ The treatment of polycystic lung mainly includes preventing lung infections, enhancing immunity, improving the living environment, and keeping the air fresh. If respiratory failure and renal failure occur, the most effective treatment is a combined lung and kidney transplantation.

The therapies for PLD can be roughly divided into medical and surgical strategies, and for the with the latter being the main treatment alternative despite the high cost, and the occurrence of postoperative complications and relapse. Unfortunately, our patient was considered to be unable to tolerate surgery due to the presence of numerous diffuse hepatic and renal cysts and renal failure. In recent years, blood purification has been applied to treat hyperbilirubinemia when medical and surgical treatments fail. These include bilirubin adsorption, plasma exchange, hemoperfusion, hemodialysis, hemodiafiltration, and molecular adsorbent recycling systems. Bilirubin has a molecular weight of 584.67 Da. Clinical studies have shown that hemodialysis, hemoperfusion, or hemodiafiltration combined with plasma exchange can provide good clearance of substances with medium molecular weights.^[16]^ Recent studies have suggested that the bilirubin clearance rate is significantly greater after combined CVVH-bilirubin adsorption than after plasma exchange or other blood-purification methods.^[17,18]^ Furthermore, CVVH has the advantage of regulating the acid-base balance, water and electrolyte balance, and increasing the clearance of water-soluble toxins, and is thus especially suited for critically ill patients with renal failure.^[19,20]^

## 4 Conclusion

PLD is a rare genetic anomaly. It is usually accompanied with autosomal-dominant PKD and only infrequently with polycystic lung. Nevertheless, imageological examination of the lungs is necessary for patients with PLD. Although infrequent, jaundice can occur in this group of patients and lead to severe hyperbilirubinemia. Additionally, when liver transplantation and other surgical methods are contraindicated, blood purification may be considered an alternative treatment for patients with obstructive jaundice related to PLD.

## Author contributions

**Conceptualization:** Santao Ou, Qi Liu.

**Data curation:** Santao Ou, Jiaru Lin, Ying Li, Linwang Gan

**Formal analysis:** Santao Ou.

**Writing – review & editing:** Liling Zhang.
